# Immune checkpoint inhibitors in HIV infection: current evidence on viral reservoir regulation and immune restoration

**DOI:** 10.3389/fimmu.2026.1879479

**Published:** 2026-07-22

**Authors:** Yixuan Wang, Xin Huang, Liyuan Zheng, Ling Luo, Taisheng Li, Wei Cao

**Affiliations:** Department of Infectious Diseases, Peking Union Medical College Hospital, Chinese Academy of Medical Sciences and Peking Union Medical College, Beijing, China

**Keywords:** HIV, immune checkpoint inhibitors, PD-1/PD-L1, viral reservoir, immune restoration, T-cell exhaustion

## Abstract

Immune checkpoint inhibitors (ICIs), particularly those targeting the programmed cell death-1 (PD-1)/programmed cell death-ligand 1 (PD-L1) axis, have emerged as potential tools beyond cancer immunotherapy in people with HIV (PWH). In the setting of effective antiretroviral therapy (ART), chronic inflammation, T-cell exhaustion, and persistent viral reservoirs remain major obstacles to durable HIV remission or cure. Accumulating evidence suggests that ICIs may modulate these processes by reversing latency, enhancing HIV-specific immune responses, and contributing to reservoir regulation and immune restoration. In this review, we summarize current evidence on the role of ICIs in HIV infection, with a focus on reservoir regulation and immune restoration. We discuss the biological rationale for checkpoint blockade in chronic HIV infection and review available data from preclinical models, clinical studies in PWH with cancer, exploratory trials in non-cancer populations, and combination cure strategies. We also address recent findings on proviral landscape changes and key safety considerations. Overall, ICIs represent a promising but still limited component of HIV cure-oriented strategies. Their effects on reservoir dynamics and immune restoration remain heterogeneous, and further progress will require safer dosing approaches, biomarker-guided patient selection, standardized reservoir assessment, and well-designed clinical trials.

The success of modern antiretroviral therapy (ART) has greatly changed the scenario of human immunodeficiency virus (HIV)-1 infection. With sustainable suppression of viral replication and adequate immune reconstitution, people with HIV (PWH) are able to achieve life expectancy comparable with seronegative people. As a result, non-AIDS-defining conditions (NADCs) not directly related to immunodeficiency become a non-negligible contributor to the outcome of PWH. These include cardiovascular and metabolic disorders, renal impairment, osteoporosis, neuropsychological diseases, and non-AIDS-related malignancies such as lung cancers, melanoma, gastrointestinal tumors, among others. It is currently widely accepted that increased risks of these comorbidities in PWH are partially rooted in the persistence of latent viral reservoir and unresolved chronic inflammation, which, despite long-term effective ART, still presents a major challenge to HIV care (1, 2).

Immune checkpoint inhibitors (ICIs) targeting the programmed cell death-1 (PD-1) pathway are a class of anti-cancer immunomodulatory agents that have altered the therapeutic paradigms of many cancers, including types that occur at higher rates in PWH than in the general population. Although PWH were usually excluded from most initial clinical trials of these agents due to safety concerns (3), preliminary performance from case series, small cohorts, systematic reviews, and recent real-world data has been reported and reviewed (4–10). Based on available evidence, ICIs targeting PD-1 or its ligand PD-L1 were generally well tolerated and safe for PWH with cancers of indication, though responses to ICIs were variable between individuals, indicating that controlled clinical trials with larger sample size are needed. Nevertheless, according to current studies, levels of plasma viral load measured by HIV RNA and CD4+ T cell counts remained mostly unaffected over the application of ICIs (7, 8, 10).

Interestingly, immune checkpoints and their downstream pathways are not only a critical player in the microenvironment of tumor pathogenesis, but are also involved in the immune interactions between HIV and the host, as T cell exhaustion resulted from persistent inflammation is a common feature shared by cancer and many chronic infections including HIV infection (11, 12). It has been hypothesized that modulation of immune checkpoints through ICIs may also have a positive effect on the control of residual inflammation and viral reservoirs in HIV infection, which may present a potential approach for further viral eradication. Therefore, in addition to cancer responses, changes of virological and immunological profiles following ICIs are quite appealing in terms of HIV control itself. Up to date, many ICIs have been used in PWH with indicated cancers, and some studies also paid special attention to their impact on HIV clearance. Here, we reviewed evidence of ICI performance in PWH regarding virological and immune restoration, so as to provide more understanding of HIV immunopathogenesis and further insights into HIV cure.

## HIV infection: role of immune checkpoints

HIV infection causes continuous damage and depletion of CD4+ T cells in the majority of untreated individuals. Although virus-specific T cell responses could be evoked in the initial phase and exert some cytotoxic antiviral effect, with prolonged viremia and persistent immune activation, inhibitory and exhausted phenotypes begin to accumulate in virus-specific T cells. Even in the setting of well-controlled HIV infection with normal or near-normal CD4+ T cell counts, persistence of chronic viral antigenemia may lead to a generalized upregulation of immune checkpoint proteins on CD4+ and CD8+ T cells, leading to a reduced capacity to produce cytokines and effector molecules (13).

Common inhibitory proteins usually include PD-1, cytotoxic T lymphocyte antigen-4 (CTLA-4), LAG-3 (lymphocyte activation gene protein 3), and TIM-3 (T-cell immunoglobulin domain and mucin domain-containing protein 3), among others. In acute infections, immune checkpoint proteins are transiently upregulated on the surface of effector T cells following T-cell activation, mainly to attenuate T-cell activation and limit immune reactions by end of an acute infection to avoid undue immune damage. However, during chronic infection or cancer, presence of persistent foreign antigens leads to over-stimulation of T cells, which become exhausted with increasing loss of effector function and increased expression levels of immune checkpoint proteins (14, 15).

PD-1 acts as a master inhibitory regulator of T cell function together with others, and plays a critical role in balancing pathogen elimination and histopathological damage. PD-1 is expressed by activated T cells, B cells, natural killer (NK) cells, dendritic cells, and possibly activated monocytes. It interacts with two ligands PD-L1 and PD-L2. PD-L1 is mainly expressed on hematopoietic cells, including T and B cells, dendritic cells, macrophages, and constitutively on some epithelial and endothelial cells. In contrast, expression of PD-L2 is more restricted on antigen presenting cells including dendritic cells, macrophages, mast cells, and some B cells. The interaction of PD-1 and its ligands regulates T cell receptor signaling, leading to distinct functional, transcriptional and metabolic changes, and finally diminished effector T cell functions, including cytokine secretion, proliferation, cytotoxicity, motility, and cell survival (15).

In chronic HIV infection, persistence of viral antigen and associated pro-inflammatory status contribute to T cell exhaustion, among which PD-1 expression on virus-specific T cells is a primary marker correlated with disease progression. It has been reported that expression of PD-1 is significantly correlated with both the viral load and reduced capacity for cytokine production and proliferation of specific CD8+ T cells (13, 16). Meanwhile, exhausted virus-specific CD4+ T cells also express elevated levels of PD-1 correlating with disease progression, viral load and reduced CD4+ T-cell count (17). High expression of PD-1 was observed in the germinal center of lymph nodes in PWH, mainly corresponding to the CD4+ T follicular helper (Tfh) cells (18). Following effective ART, levels of PD-1 gradually decrease on HIV-specific CD4+ and CD8+ T cells, though still remain elevated compared with HIV seronegative people (13). Such trend of exhaustion of HIV-specific T cells is established in early infection, and presents an almost insurmountable barrier to the host-mediated elimination of HIV.

**Figure 1:**
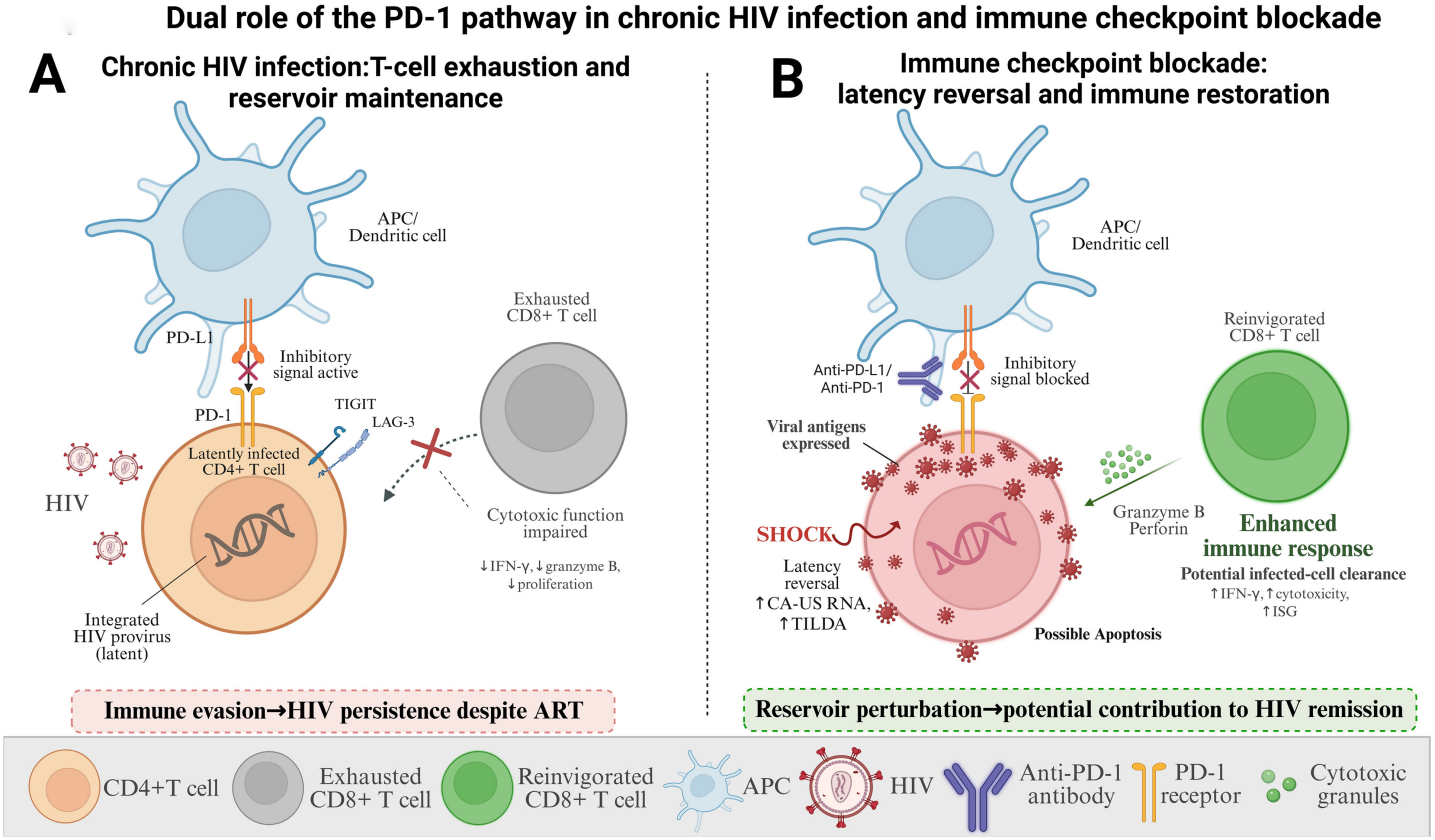
Dual role of the PD-1 pathway in chronic HIV infection and immune checkpoint blockade. **(A)** Chronic HIV infection: persistent antigenemia drives upregulation of PD-1, TIGIT, and LAG-3 on virus-specific T cells. PD-1 engagement with PD-L1 on antigen-presenting cells delivers inhibitory signals, leading to CD8+ T-cell exhaustion (diminished IFN-γ, reduced cytotoxicity, impaired proliferation) and continued HIV persistence despite ART. **(B)** Immune checkpoint blockade: anti-PD-1/PD-L1 antibodies block the PD-1:PD-L1 interaction, producing a dual effect. In latently infected CD4+ T cells, transcriptional reactivation leads to viral antigen expression and increased CA-US RNA (“shock”). Concurrently, exhausted HIV-specific CD8+ T cells regain effector functions including IFN-γ production and cytotoxic activity (“kill”), driving reactivated cells toward immune-mediated clearance.APC, antigen-presenting cell; ART, antiretroviral therapy; CA-US RNA, cell-associated unspliced HIV RNA; CTLA-4, cytotoxic T-lymphocyte-associated antigen 4; IFN-γ, interferon-gamma; ISG, interferon-stimulated gene; LAG-3, lymphocyte activation gene 3; PD-1, programmed cell death-1; PD-L1, programmed cell death-ligand 1; TIGIT, T-cell immunoreceptor with immunoglobulin and ITIM domains. Created with BioRender.com.

On the other hand, multiple observational studies also demonstrated a clear association between expression of immune checkpoints and the size of the HIV reservoir. More specifically, CD4+ T cells co-expressing PD-1, TIGIT, and LAG-3 are major cellular hosts of persistent HIV during ART, with triple-positive cells enriched 8.2-fold for integrated HIV DNA compared with bulk CD4+ T cells, and cells expressing at least one of these checkpoint molecules accounting for a median of 76% of the inducible reservoir (19). Subsequent studies have further revealed its functional significance: PD-1 not only marks HIV reservoirs, but also actively suppresses the virus at the transcriptional level, maintaining its latent state. Mechanistically, activation of PD-1 suppresses HIV transcription (by reducing LTR activity and inhibiting TCR-induced viral reactivation); conversely, in CD4+ T cells from PWH on ART (ART), the ex vivo application of PD-1 blockade in combination with a latency reverser significantly enhances HIV production without inducing systemic T-cell activation (20). This indicates that PD-1+ CD4+ T cells not only harbor viral reservoirs, but that PD-1 blockade can lift the suppression of viral transcription, providing direct mechanistic evidence for the use of ICIs as latency-reversing agents. Furthermore, HIV DNA and unspliced RNA were found to be enriched in PD-1+ cells residing in both peripheral blood and lymph nodes of PWH on suppressive ART, establishing a direct mechanistic link between immune checkpoint expression and viral persistence (18–20). It is postulated that modulation of the PD-1 pathway may lead to better virologic control by promoting CD8+ T-cell expansion and augment HIV-specific CD4+ and CD8+ T cell function (13, 16, 19, 21). The dual role of the PD-1 pathway in driving T-cell exhaustion during chronic HIV infection and its potential reversal through immune checkpoint blockade is illustrated in **Figure 1**.

## Impact of ICIs on HIV persistence

Although PWH are not considered an ideal population for studies of ICIs in cancer treatment, case reports and retrospective cohort studies have been reported over the recent ten years, including the application of ICIs targeting PD-1, PD-L1 or CTLA-4 in people with HIV with lung cancer, melanoma, head and neck cancer, etc. Specifically, use of nivolumab, pembrolizumab, ipilimumab, and durvalumab, or combinations thereof have been reported in PWH. Based on current evidence, ICIs were generally well-tolerated and safe for PWH with comparable efficacy. A systematic review revealed that the overall response and adverse event rates of ICIs in PWH were similar to the general population (8). Nevertheless, in reported cases with virological assessment, the viral load and CD4+ levels in most PWH receiving ICIs were stable.

**Figure 2:**
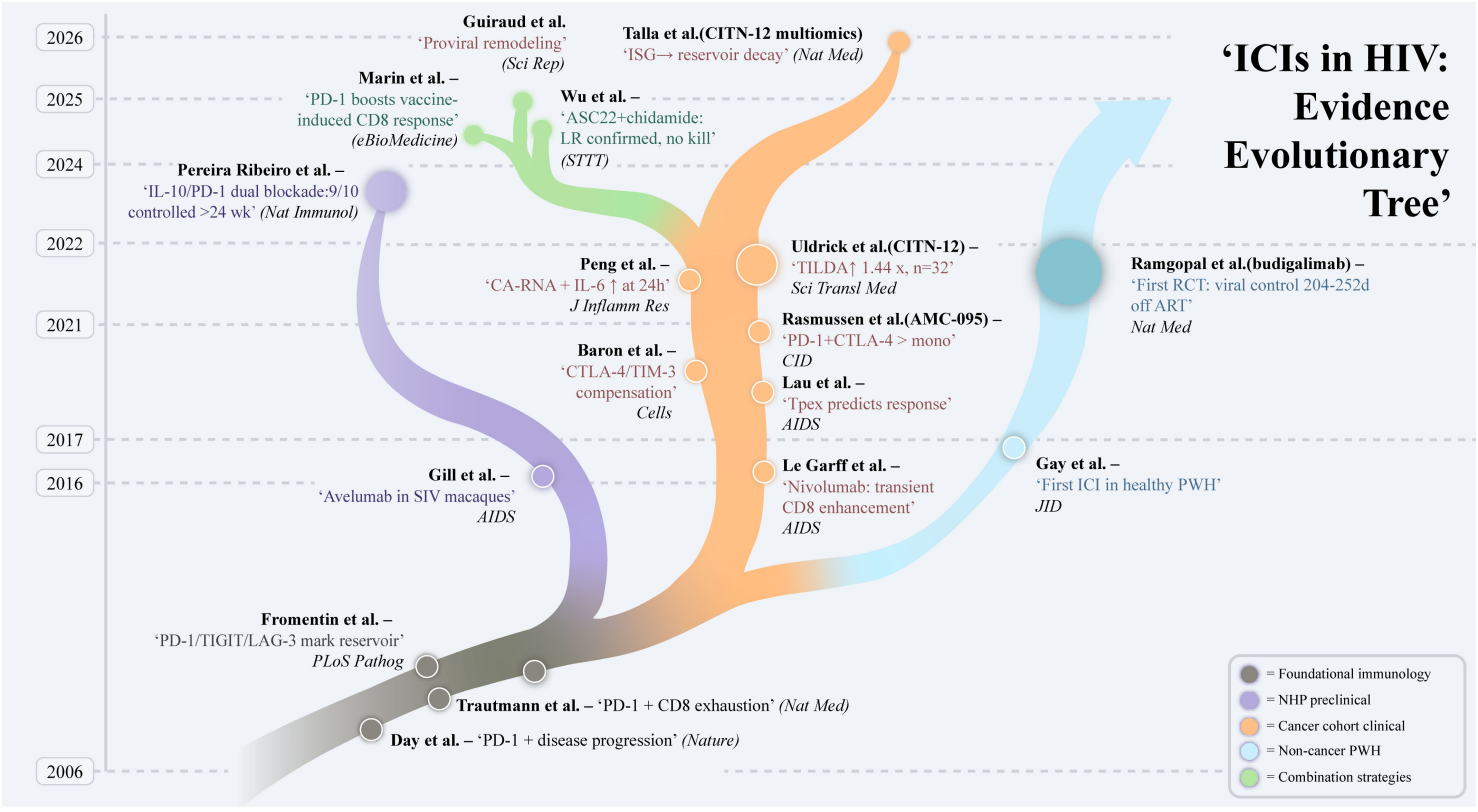
Timeline of key studies evaluating immune checkpoint inhibitors in HIV infection. The image presents the chronological development of evidence on ICIs in HIV research from 2006 to 2026, visualized as an evolutionary tree in which a single trunk of foundational studies diverges into multiple branches as the field matures. Studies are categorized into five streams, each represented by a distinct color: foundational immunology studies establishing the role of immune checkpoints in HIV persistence (grey), nonhuman primate preclinical studies (purple), cancer cohort clinical studies (orange), studies in non-cancer PWH (blue), and combination and proviral-landscape studies (green). Each entry indicates the first author, principal finding, and journal of publication. ART, antiretroviral therapy; ATI, analytical treatment interruption; CA-RNA, cell-associated HIV RNA; ICIs, immune checkpoint inhibitors; ISG, interferon-stimulated gene; LR, latency reversal; NHP, nonhuman primate; PWH, people with HIV; RCT, randomized controlled trial; TILDA, Tat/Rev-induced limiting dilution assay; Tpex, progenitor exhausted T cells.

As mentioned, PD-1 and CTLA-4 expression are increased in the setting of chronic HIV infection, and HIV DNA and unspliced RNA are enriched in PD-1+ cells in blood and lymph nodes of PWH even following effective ART. Therefore, it would be very inspiring to learn how the viral reservoir and related immune responses react upon ICI use in these individuals. Some studies have documented transient increases in HIV transcription in CD4+ T cells upon treatment of anti-PD-(L)1 drugs, although many of these participants later experienced decreases in plasma HIV RNA. The following sections systematically review current evidence regarding the effects of ICIs on HIV persistence, encompassing preclinical animal models, clinical observations in PWH with cancer, exploratory studies in non-cancer populations, and combination cure strategies (19–24). **Figure 2** presents a chronological overview of these key studies by experimental setting,and **Table 1** and **2** provide detailed information on virological and immunological outcomes (**Table 1**) and study design, methodological characteristics, and key limitations (**Table 2**).

**Table 1:** Key studies of immune checkpoint blockade or checkpoint-targeted strategies in HIV reservoir regulation and immune restoration.

Study	Type	N	Population	ICI/checkpoint strategy	CA-US RNA/latency reversal	HIV DNA/reservoir	Plasma RNA/ATI outcome	Key immunological findings	Assay
Mechanistic and ex vivo checkpoint−targeting studies									
Fromentin 2016 (19)	Reservoir−marker study	48	ART−suppressed PWH	Checkpoint profiling (PD−1, TIGIT, LAG−3)	CA−US RNA measured; no blockade	PD−1/TIGIT/LAG−3 associated with integrated HIV DNA; triple−positive cells 8.2−fold enriched	Not assessed	Cells expressing >=1 marker contributed median 76% ofinducible reservoir	Total/int. DNA, 2−LTR, CA−US RNA, TILDA
Fromentin 2019 (20)	Ex vivo latency study	4−8 donors	ART−suppressed PWH samples	Pembrolizumab + bryostatin ex vivo	PD−1 engagement inhibited reactivation; blockade increased HIV production	PD-1 cells enriched for integrated HIV DNA/reservoir	Not assessed	Latency reversal potentiated without global T−cell activation	p24, RT−PCR, flow cytometry
Preclinical studies									
Gill 2016 (18)	Nonhuman primate study	6	SIV−infected RM on ART	Avelumab (anti−PD−L1)	NR	NR	Trend down (NS)	No significant CD4/CD8 changes	Plasma VL
Pereira Ribeiro 2024 (25)	Nonhuman primate study	28	SIV−infected RM on ART	Anti−IL−10 + anti−PD−1	Lower LN CA−vRNA/2−LTRs post−ATI with combo	Lower LN CA−vDNA/IPDA vs controls/IL−10 at 24 wk post− ATI	9/10 combo >24 wk control	Increased CD8 effector cells in LN; reduced BCL−2 in CD4; TCF−1+ memory cells	Plasma VL, IPDA/2−LTR, CA−vRNA/CA−vDNA, flow cytometry
Clinical studies in PWH with cancer									
Le Garff 2017 (26)	Case report	1	HIV + NSCLC	Nivolumab (anti−PD−1)	NR	Mild increase	Stable	Increased HIV−specific IFN−γ+ CD8; reduced PD−1; increased IL−6	CA−HIV DNA
Peng 2022 (27)	Case series	3	HIV + NSCLC	Anti−PD−1	Increased CA−HIV RNA (P=0.049)	Increased HIV DNA (P=0.042)	Stable	IL−6 increased and correlated with HIV DNA	CA−HIV RNA, HIV DNA
Uldrick 2022 (22) (CITN−12)	Prospective virologic substudy	32	HIV + advanced cancer	Pembrolizumab (anti−PD−1)	Increased CA−US RNA/RNA : DNA ratio; TILDA 1.44−fold at C6 (P=0.008)	Transient day−2 HIV DNA decrease; returned to baseline by day 22	Generally stable	CD4:CD8 ≥0.5 predicted latency reversal	TILDA, CA−US RNA, HIV DNA
Baron 2022 (28)	Prospective observational cohort	32	HIV + cancer	Anti−PD−1	NR	CA−HIV DNA tended to decrease before cycle 2; >=0.5 log10 decrease in 6 patients	No HIV virologic failure signal emphasized	No overall HIV−specific immunity increase; early CTLA−4+ CD4 expansion; compensatory TIM−3/CTLA−4 signals	CA−HIV DNA, viral loads, flow cytometry
Talla 2026 (29) (CITN−12 multiomics)	Prospective multiomic substudy	30	HIV + cancer (CITN−12)	Pembrolizumab (anti−PD−1)	Increased transcriptional activation markers	2.39−fold HIV DNA reduction in ISG−high EOT subset	Transient plasma HIV RNA increase; no ATI	Increased HIV−specific CD8 at 24 h; reduced TGF−β; ISG/TLR signatures linked to reservoir decay	Multiomic, TILDA
Rasmussen 2021 (23) (AMC−095)	Prospective virologic substudy of a phase I trial	40	HIV + solid tumor/cHL	Nivolumab +/− ipilimumab	Increase with combination only	Small total HIV DNA decrease across first 4 cycles; QVOA decreased 97%/64% in 2 combo participants	Modest transient increase in plasma HIV RNA at day 7; no ATI	NR; no dedicated HIV-specific immune-response analysis was reported	CA−US RNA, HIV DNA, QVOA
Lau 2021 (30)	Case series	3	HIV + cancer	Anti−PD−1/PD−L1 +/− anti−CTLA−4	CA−US RNA increased after each infusion in all 3	No consistent change; P3 had HIV DNA/TILDA decreases	Stable	P2 had strongest HIV−specific CD8 response and highest pre−ICB Ki67+ Tpex	CA−US RNA, HIV DNA
Clinical studies in PWH without cancer									
Gay 2017 (32)	Randomized phase I trial	8	HIV only (no cancer)	BMS−936559 (anti−PD−L1)	No change	No change	Stable	2/6 increased HIV−specific CD8 (IFN−γ, CD107a)	Plasma RNA, CA−RNA, CA−DNA
Gay 2024 (33)	Randomized phase I/II trial	5	HIV only (no cancer)	Cemiplimab (anti−PD−1)	Transient CA−RNA/plasma RNA increase in one responder	Total HIV DNA decreased in one responder; IPDA difficult to interpret	No virologic failure	One 2−dose recipient had 6.2−fold CD8 and 3.4−fold CD4 polyfunctional Gag responses	CA−RNA, total DNA, IPDA, SCA, flow cytometry
Budigalimab 2025 (34)	Randomized phase Ib trial	41	HIV only (no cancer)	Budigalimab (anti−PD−1)	NR	NR	6/11 (6/9 completers) delayed rebound/control; 2 ART−free to 204/252 d	Exploratory post−ATI viral−control signal; requires validation	Plasma VL post−ATI
ASC22 + chidamide 2024 (24)	Phase II single−arm study	15	HIV only (no cancer)	ASC22 anti−PD−L1 + chidamide	Increased at wk 8 (P<0.05)	Transient increase at wk 4 (P=0.014); integrated DNA unchanged	Stable	No significant HIV−specific CD8 increase; effector-memory T-cell subsets increased	CA−HIV RNA, total/int. DNA
Therapeutic−vaccine and proviral−landscape analyses									
Guiraud 2025 (39)	Proviral−landscape study	3	HIV + cancer (OncoVIHAC); suppressed >=13 y	Anti−PD−1, anti−PD−L1, or anti−PD−1/CTLA−4	Not assessed	No significant total CA−HIV DNA change; no significant integration−site/epigenetic shift; PD−1/CTLA−4 case lost NIN clone (17% pre)	No ATI; no HIV blips during ICI	Post−ICI lower pol frameshifts; PD−1 or PD−1/CTLA−4 linked to gag frameshifts and more STOP codons; limited diversity reduction	MIP−seq, ISLA, nFGS, total HIV DNA
Marin 2026 (35)	Ex vivo vaccine−sample study	33	Etvac 11; Et 13; Chro 9	PD−1 or TIM−3 blockade ex vivo	Not assessed	Not assessed	Not assessed	PD−1 blockade enhanced vaccine−induced HIV−specific CD8 responses; TIM−3 blockade had no effect	Flow cytometry, multiplex assays, p24

ART, antiretroviral therapy; ATI, analytical treatment interruption; CA−US RNA, cell−associated unspliced HIV RNA; CA−HIV RNA, cell−associated HIV RNA; cHL, classical Hodgkin lymphoma; ICI, immune checkpoint inhibitor; IL, interleukin; IPDA, intact proviral DNA assay; ISG, interferon−stimulated gene; ISLA, integrationsite and library amplification; MIP−seq, matched integration site and proviral sequencing; nFGS, near full genome sequencing; LN, lymph node; NR, not reported; NS, not significant; PWH, people with HIV; QVOA, quantitative viral outgrowth assay; RM, rhesus macaque; SCA, single−copy assay; TCF−1, T cell factor 1; TGF−β, transforming growth factorbeta; TILDA, Tat/Rev−induced limiting dilution assay; Tpex, progenitor exhausted T cells; VL, viral load; wk, weeks; int., integrated; seq., sequencing. References in square brackets correspond to the 50−reference main manuscript.

**Table 2:** Study design, methodological characteristics, and key limitations of the included studies.

Study	Control/comparator	ICI dose & schedule	ART suppression criteria	ATI	Primary endpoint(s)	Follow-up	Safety outcome	IPDA	Tissue samples	Key limitations
Mechanistic and ex vivo checkpoint−targeting studies										
Fromentin 2016 (19)	Marker−negative and marker−positive subsets	No ICI; checkpoint phenotyping and cellsorting	ART >3 y; CD4 >350; HIV RNA <40 copies/mL	No	Associations between checkpoint expression and HIV persistence markers	Cross−sectional	Not applicable	No	No (blood/PBMC)	Cross−sectional; multiple comparisons; mostly DNA endpoints; tissue reservoirs notassessed
Fromentin 2019 (20)	Isotype control and PD−L1 engagement conditions	Pembrolizumab 10 microg/mL ex vivo +/− bryostatin 10 nM	ART−suppressed PWH samples	No	Ex vivo HIV production/latency reversal and T−cell activation	3−6 d cultures	Not applicable	No	Yes (lymph node and gut biopsies)	Ex vivo only; small donor sets; in vivo efficacy and toxicity not tested
Preclinical studies										
Gill 2016 (18)	Yes (saline 3; avelumab 3)	Avelumab 20 mg/kg IV weekly x24 wk	SIV−infected RM, cART−suppressed	Yes	PD−1/PD−L1 expression; safety; SIV rebound	24 wk + ~10 wk post−ATI	Well tolerated; no significant clinical or laboratory toxicity observed.	No	Yes (LN, spleen)	NHP n=6; prior IL−15; rebound non−significant; short follow−up
Pereira Ribeiro 2024 (25)	Yes (vehicle 8; Anti-IL-10 10; combo 10)	Anti−IL−10 10 mg/kg +/− anti−PD−1 10 mg/kg IV, q3−4wk	ART from wk 6 post−infection, ~14 mo	Yes	Post−ATI rebound control; CA−vRNA/CA−vDNA, IPDA/2−LTR and immune correlates	To 24 wk post−ATI	1 IL−10 + 1 combo severe AE (euthanized)	Yes	Yes (blood + LN)	NHP/SIV; small n; no anti−PD−1−only arm; controls euthanized at 24 wk; human data lacking
Clinical studies in PWH with cancer										
Le Garff 2017 (26)	No (single case)	Nivolumab x7 (dose NR)	Suppressed; ABC/3TC/DTG	No	Immuno−virologic dynamics	To D120	Grade I hepatotoxicity	No	No (blood)	Single case; cancer/therapy confounders; dose NR
Peng 2022 (27)	No (3 cases)	Anti−PD−1 x14 q21d + chemotherapy	Chronic ART; 2 undetectable, 1 = 73 copies/mL	No	CA−HIV RNA, HIV DNA,cytokines per infusion	~39−42 wk	Safe/well tolerated in 3; AE details limited	No	No (PBMC)	n=3; mixed anti−PD−1 + chemotherapy; no long−term data
Uldrick 2022 (22) (CITN−12)	No (single−arm)	Pembrolizumab 200 mg IV q3w, <=2 y	HIV RNA <200; CD4 >100; most <20 copies/mL	No	Latency reversal and persistence	Up to 2 y; EOT + 30 d	Mostly undetectable HIV; 1 low−level viremia	No	No (blood)	No control; no IPDA/QVOA; EOT confounded; no reservoir clearanceshown
Baron 2022 (28)	No (observational cohort)	Anti−PD−1 per cancer protocol	On ART; VL <50 or <200 copies/mL according to cohort	No	CA−HIV DNA, plasma/other viral loads, checkpoint and virus−specific T cells	Median 5 mo	1 irAE death reported; no HIV virologic failure signal emphasized	No	No (blood)	Observational; heterogeneous cancers and regimens; compensatory checkpoint changes; no randomized control
Talla 2026 (29) (CITN−12 multiomics)	No (single−arm)	Pembrolizumab 200 mg IV q3w, <=2 y	Suppressed; ART >=4 wk	No	Immune multiomic signatures vs reservoir decay	EOT 44−315 d; <=2 y	Per CITN−12, mostly grade 1−2	No	No (blood)	Exploratory; small n; no control; ISG strata observational; no IPDA/QVOA
Rasmussen 2021 (23) (AMC−095)	No placebo; monotherapy vscombination	Nivolumab 3 mg/kg or 240 mg q2w +/− ipilimumab 1 mg/kg q6w	HIV RNA <LLOD for 4 wk	No	CA−US RNA, HIV DNA, plasma RNA, QVOA	2−35 wk (mean 20.6)	Per parent trial	No	No (blood)	No randomized control; combo n=7; QVOA paired n=10; cancer confounders
Lau 2021 (30)	No (case series)	Avelumab 10 mg/kg q2w; nivolumab 3 mg/kg + ipilimumab 1 mg/kg q3w	Suppressed <20 copies/mL for 9−11 y	No	Virologic + HIV−specific T−cell changes	First 4 infusions + 8 wk	No irAE in any participant	No	No (blood)	n=3; descriptive only; mixed regimens;cancer confounders; bloodonly
Clinical studies in PWH without cancer										
Gay 2017 (32)	Yes (drug 6; placebo 2)	BMS−936559 0.3 mg/kg IV single dose	CD4 >=350; HIV RNA <40 copies/mL	No	Safety; D28 Gag−specific CD8	48 wk	1 hypophysitis (resolved); no grade >=3 related AE	No	No (blood)	Very small; single low dose; escalation halted; no reservoir effect
Gay 2024 (33)	Yes (4:1 randomized; cemiplimab 4, placebo 1)	Cemiplimab 0.3 mg/kg IV at wk 0/6 planned; 1−2 doses received	ART >=24 mo; CD4 >=350; HIV RNA <50 copies/mL	No	Safety; HIV−specific polyfunctional CD4/CD8 responses; virologic assays	48 wk	Grade 2 thyroiditis and possible grade 3 hepatitis after one low dose; study stopped	Yes	No (blood/PBMC)	Early closure; n=5; only lowest dose tested; one placebo participant
Budigalimab 2025 (34)	Yes (placebo−controlled)	Budigalimab 2 or 10 mg IV q4w x2, or 10 mg q2w x4	HIV RNA <20 copies/mL>=6 mo; CD4 >=500	Yes	Safety, tolerability, PK; exploratory control	Stage I 44 wk; Stage II 36 wk	Mostly grade 1−2; 2 grade 1 irAE; no deaths	No	Yes (rectal biopsy)	Exploratory; small n; ATIconfounds drug effect; needs phase 2
ASC22 + chidamide 2024 (24)	No (single−arm)	ASC22 1 mg/kg SC q4w x3 + chidamide 10 mg PO 2x/wk x12 wk	Suppressed; all >=2 y stable	No	PBMC total/integrated HIV DNA to wk 24	12 wk + 12 wk	9 grade 1 AEs, self−resolved	No	No (PBMC)	Single−arm; n=15; no ATI/IPDA; cannot separate two drugs
Therapeutic−vaccine and proviral−landscape analyses										
Guiraud 2025 (38)	Pre−post within−patient; no external control	ICI per cancer protocol: anti−PD−1, anti−PD−L1, or anti−PD−1/CTLA−4	Virologically suppressed >=10 y;all >=13 y in analysis	No	MIP−seq integration−siteand proviral−sequence landscape; total CA−HIV DNA	Pre first infusion; post >=2 mo after last ICI cycle	Selected patients had no major treatment side effects; no HIV blips	No	No (blood/PBMC)	n=3; one per regimen; cancer confounding; limited material; subtype B only; blood only; no lymphoid tissue; epigenetic context inferred from databases
Marin 2026 (35)	Etvac 11, Et 13, Chro 9 in ICB assay	Anti−PD−1 10 microg/mL +/− anti−TIM−3 5 microg/mL ex vivo	Etvac/Et early ART; Chro ART >4 y, VL <50 copies/mL	No	Functional vaccine−induced and HIV−specific CD8 responses	6−7 d ex vivo assay	Not applicable	No	No (blood)	Retrospective ex vivo; limited sample size/few women; different antigens across groups; no clinical dosing or reservoir endpoint

ART, antiretroviral therapy; ATI, analytical treatment interruption; AE, adverse event; ABC, abacavir; BMS−936559, anti−PD−L1 antibody; CA−US RNA, cell−associated unspliced HIV RNA; cART, combination ART; DTG, dolutegravir; EOT, end of treatment; ICI, immune checkpoint inhibitor; IPDA, intact proviral DNA assay; irAE, immune−related adverse event; IV, intravenous; LLOD, lower limit of detection; MIP−seq, matched integration site and proviral sequencing; LN, lymph node; NHP, non−human primate; NR, not reported; PBMC, peripheral blood mononuclear cell; PK, pharmacokinetics; PO, by mouth; PWH, people with HIV; q2w/q3w/q4w/q6w, every 2/3/4/6 weeks; QVOA, quantitative viral outgrowth assay; RM, rhesus macaque; SC, subcutaneous; SIV, simian immunodeficiency virus; 3TC, lamivudine. References in square brackets correspond to the 50−reference main manuscri

### Preclinical evidence from nonhuman primate models

Prior to clinical evaluation in humans, the rationale for employing immune checkpoint blockade (ICB) to perturb HIV persistence was first tested in nonhuman primate (NHP) models of simian immunodeficiency virus (SIV) infection. Notably, PD-1 is not only upregulated on CD4+ and CD8+ T cells during chronic SIV infection, but is also highly expressed on cells harboring latent reservoirs. In an early proof-of-concept study, Gill et al. characterized the distribution of PD-1/PD-L1 in lymph nodes of PWH and demonstrated that PD-1/PD-L1 expression did not correlate with viremia levels (18). In a subsequent chronic SIV-infected rhesus macaque model, the anti-PD-L1 monoclonal antibody avelumab was administered, which was generally well tolerated and exhibited a transient trend toward viral control following analytical treatment interruption (ATI), although statistical significance was not achieved. Despite the inconclusive virological outcome, this study provided an early proof-of-concept that PD-L1 blockade could potentially modulate post-ART viral dynamics and identified the PD-1/PD-L1 axis as a potential target for reservoir intervention and prolonged viral control.

A considerably more impactful NHP study was subsequently reported by Pereira Ribeiro and colleagues in 2024 (25). Recognizing that HIV/SIV persistence during ART is associated not only with elevated PD-1 expression but also with heightened plasma interleukin-10 (IL-10) levels, the investigators hypothesized that dual blockade of these two immunosuppressive pathways might achieve synergistic effects. Twenty-eight ART-treated SIV*mac*239-infected rhesus macaques were assigned to receive anti-IL-10 alone (n=10), anti-IL-10 plus anti-PD-1 (combination, n=10), or vehicle control (n=8), with ATI initiated after 12 weeks of immunotherapy. Strikingly, durable control of viral rebound was observed in nine of the ten combination-treated animals for more than 24 weeks post-ATI, whereas neither single-agent anti-IL-10 nor vehicle achieved comparable control. Mechanistic analyses revealed that several pre-ATI parameters were predictive of subsequent viral control, including induction of inflammatory cytokines, proliferation of effector CD8+ T cells within lymph nodes, and reduced expression of the anti-apoptotic molecule BCL-2 in CD4+ T cells. The underlying mechanisms likely involve remodeling of the inflammatory and chemokine microenvironment to favor viral control, enhanced early CD8+ T cell proliferation and function following ATI, and subsequent establishment of TCF-1-positive memory stem-like T cells alongside stronger coordinated CD4+/CD8+ antigen-specific immune responses in the later phase. Although the overall safety profile of the combination regimen was acceptable, it was not entirely devoid of immune-related adverse effects. These findings represented a major preclinical advance, providing compelling evidence that multi-pathway immunomodulatory strategies targeting both innate and adaptive immune suppression could achieve sustained virological remission in a stringent NHP model, and offering a strong rationale for clinical translation. However, whether similar efficacy and safety can be achieved in humans, the optimal dosing schedule and timing of intervention, and the potential needs for integration with other cure strategies, all remain to be determined in future studies.

### Clinical observations on HIV persistence in PWH receiving ICIs for cancer

Investigators began to evaluate the effects of ICIs on HIV reservoirs and immune function first in PWH receiving these agents for concurrent malignancies. Multiple studies have reported a recurring pattern: transient increases in cell-associated unspliced HIV RNA (CA-US RNA), variable changes in plasma HIV RNA, compensatory checkpoint remodeling, and in some cases modest declines in total HIV DNA, collectively suggesting that ICIs may exert a latency-reversing effect while potentially facilitating partial clearance of infected cells (26–31).

Individual case observations. Le Garff and colleagues (26) reported the case of a 53-year-old man with HIV and non-small cell lung cancer (NSCLC) who received seven cycles of nivolumab. During treatment, plasma HIV viral load remained stable, while HIV-specific interferon-gamma (IFN-γ)-producing CD8+ T cells were modestly elevated, particularly within the transitional memory subset, accompanied by a decline in T cell surface PD-1 expression and an increase in IL-6 levels. These findings suggested that PD-1 blockade may transiently activate HIV-related immune responses and partially alleviate exhaustion phenotypes, with a mild increase in reservoir-associated DNA level, yet the actual antiviral and anti-reservoir effect of such fluctuation remained uncertain. Subsequently, Peng et al. (27) documented three PWH with NSCLC who received 14 infusions of anti-PD-1 therapy and observed that CA-HIV-RNA, HIV DNA, and IL-6 levels in peripheral blood mononuclear cells (PBMCs) were all significantly elevated within 24 hours of each infusion, suggesting early activation of the HIV reservoir with concomitant immune activation following PD-1 blockade. Still, the long-term consequences of such transient changes remained to be elucidated. It is worth noting that the elevations in CA-HIV-RNA and HIV DNA observed in the two studies described above primarily reflect latency reversal (shock) and accompanying immune changes, rather than genuine reservoir clearance (kill). In particular, in the study by Peng et al., the increase in HIV DNA was associated with elevated IL-6, suggesting that these changes may have been driven more by global immune activation than by a specific anti-reservoir effect. Baron et al., in a larger cohort of PWH with cancer, simultaneously assessed reservoir dynamics, immune responses, and safety, providing a more systematic complement to the case observations described above (28). The study found that evidence of reservoir reduction was weak and highly heterogeneous; peripheral T cells showed clear early activation, but HIV-specific CD8+ T-cell responses in peripheral blood were not markedly enhanced. More importantly, compensatory upregulation of CTLA-4 and TIM-3 was observed, and this phenomenon was more pronounced in individuals who did not exhibit reservoir reduction. This suggests that compensatory upregulation of other immune checkpoints may partially explain why single-agent PD-1 blockade fails to effectively clear the reservoir, further supporting the rationale for combination blockade of multiple checkpoint pathways.

## The CITN-12 study (pembrolizumab)

The most comprehensive prospective evaluation of ICIs on HIV persistence in the cancer setting was conducted within the Cancer Immunotherapy Trials Network 12 (CITN-12; NCT02595866), a multicenter, open-label, phase I clinical trial evaluating pembrolizumab in ART-suppressed PWH with advanced cancers (22). In a prespecified virological substudy of 32 evaluable participants receiving pembrolizumab (200 mg intravenously every 3 weeks), dynamics of HIV reservoir were assessed using the Tat/Rev-induced limiting dilution assay (TILDA), and quantification of CA HIV RNA and DNA. The study demonstrated that pembrolizumab could induce HIV latency reversal *in vivo*, as evidenced by increases in unspliced HIV RNA, RNA : DNA ratio, and TILDA measurements, although multiply spliced RNA was not significantly elevated. After six treatment cycles, the frequency of cells harboring inducible virus increased significantly to 1.44-fold above baseline (P = 0.008), before gradually returning toward pretreatment levels with continued therapy. Furthermore, among participants who completed at least seven cycles, a CD4+/CD8+ greater than 0.5 was associated with enhanced inducible viral transcription (P = 0.025), suggesting that a relatively preserved immune status may be a prerequisite for effective latency reversal. Notably, baseline CD4+ count, cancer type, or baseline HIV DNA levels did not independently predict the magnitude of reservoir perturbation. In one participant who developed persistent low-level viremia (20-400 copies/mL) after six cycles, single-genome sequencing revealed that this viremia likely originated from stochastic reactivation of diverse latent proviruses rather than selective outgrowth of a particular clone. These findings demonstrated that PD-1 blockade can indeed activate the HIV reservoir *in vivo*, supporting its candidacy as a component of HIV cure combination strategies, although current evidence has not yet supported the use of single-agent PD-1 inhibitors for meaningful reservoir induction.

Interestingly, a recent follow-up multiomic analysis of the CITN-12 cohort has provided further critical mechanistic insights (29). Longitudinal multiomic profiling of 30 participants revealed that within 24 hours of pembrolizumab infusion, there was a rapid expansion of proliferating HIV-specific effector CD8+ T cells accompanied by a decline in plasma transforming growth factor-beta (TGF-β). Among the 14 participants tracked through the end of treatment, nine displayed early and sustained induction of interferon-stimulated genes (ISGs), antiviral restriction factors, and Toll-like receptor (TLR) signaling pathways, and these individuals experienced a measurable reduction in their HIV reservoir. Importantly, the study demonstrated that anti-PD-1 therapy did not merely elicit transient fluctuations in viral transcription, but rather induced a comprehensive immune remodeling in selected individuals characterized by activation of monocyte IFN/ISG pathways, expansion of effector CD8+ T cells, enhancement of CD4+ helper responses, and downregulation of TGF-β/WNT/Treg suppressive programs. Meanwhile, the study also elucidated that only those with a favorable baseline immune status possessing intrinsic antiviral properties were likely to exhibit significant reservoir effects following ICIs. Accordingly, the potential of ICIs extends beyond mere perturbation of the viral reservoir; in select populations, these agents may foster an immune milieu conducive to HIV remission.

## The AMC-095 study (nivolumab with or without ipilimumab)

The AIDS Malignancy Consortium 095 (AMC-095) study provided the first prospective comparison of single versus combination immune checkpoint blockade in PWH with cancer (23). In this phase I trial, ART-suppressed participants with advanced solid tumors or classical Hodgkin lymphoma were assigned to receive nivolumab (anti-PD-1) with or without ipilimumab (anti-CTLA-4). A critical distinction emerged between single-agent and dual therapy: nivolumab monotherapy did not produce statistically significant changes in CA-US HIV RNA or HIV DNA, suggesting that PD-1 blockade alone may be insufficient to meaningfully reverse latency. In contrast, combination of nivolumab and ipilimumab was associated with increases in CA-US HIV RNA consistent with latency reversal, a small but statistically significant decrease in total HIV DNA, and a decline in the frequency of cells containing replication-competent virus as measured by quantitative viral outgrowth assay (QVOA) (23). In a separate report, Lau et al. also observed significant enhancement of HIV-specific CD8+ function in a subset of individuals following ICI therapy (30), and reported that pre-treatment frequency of CD8+ progenitor exhausted T cells (Tpex) appeared to correlate with this immune enhancement. These results provided the first clinical demonstration of the synergistic effect of ICIs targeting different pathways, similar to the phenomenon previously observed in animal models (25). However, the toxicity profile associated with such combination may limit its applicability in PWH without cancer.

## Heterogeneity in reported outcomes

It is important to acknowledge that findings across studies remain considerably heterogeneous. While a general trend of transient CA-US RNA elevation and HIV DNA reduction has been observed, the magnitude and duration of these changes varied substantially. Multiple factors likely contribute to this heterogeneity, including differences in patient populations (cancer type, disease stage, prior treatments), ICI regimens (PD-1 vs. PD-L1 vs. CTLA-4, monotherapy vs. combination, dosing schedules), concomitant ART regimens, and the assays used to quantify viral reservoirs (TILDA vs. QVOA vs. total HIV DNA vs. integrated HIV DNA) (31). In PWH with concurrent cancer, changes in the viral reservoir and immune function may be influenced by multiple factors whose effects are difficult to distinguish from those of ICIs. For example, the malignancy itself can suppress immunity through the tumor microenvironment and tumor burden; chemotherapy and radiotherapy can directly deplete lymphocytes, thereby exacerbating pre-existing T-cell functional exhaustion; the use of glucocorticoids and antibiotics can alter immune homeostasis; and tumor-induced cachexia and opportunistic infections can further increase the immune burden. Therefore, changes in the reservoir observed in these patients should not be simply attributed to ICIs alone. Beyond tumor-related confounders, inter-individual differences in the immune system may also be an important source of heterogeneity. For example, baseline immune status, the CD4:CD8 ratio, the frequency of precursor exhausted T cells (Tpex), reservoir composition and proviral intactness, and differences in the immune environment across tissue compartments such as peripheral blood, lymph nodes, and the gut may all influence an individual’s response to ICIs. Given the current evidence, great caution should be exercised before extrapolating reservoir changes observed in any single study of PWH with cancer to the effects of ICIs on the HIV reservoir. Therefore, researchers should prioritize rigorously controlled trials in PWH without cancer who are on suppressive ART, and limited such studies have provided preliminary findings.

### Performances of ICIs in PWH without cancer

While most data on the impact of ICIs on HIV persistence have been derived from the cancer setting, several studies have specifically evaluated ICIs in virologically suppressed PWH without malignancies. These studies are of particular importance because they eliminate the confounding effects of cancer biology and cancer-directed therapies, thereby providing more direct evidence for the potential role of ICIs in HIV cure strategies.

Gay and colleagues reported the first prospective clinical study of ICIs in otherwise healthy, ART-suppressed PWH in 2017 (32). Six participants received a single low-dose infusion of the anti-PD-L1 antibody BMS-936559 (0.3 mg/kg), with two additional participants receiving placebo. The treatment was well tolerated. Two of the six treated participants demonstrated enhanced HIV-1 Gag-specific CD8+ T cell function, as evidenced by increased production of IFN-γ, CD107a, and tumor necrosis factor. However, by day 28, no changes were detected in plasma HIV-1 RNA, CA HIV-1 RNA, or CA HIV-1 DNA. Although the absence of virological effects was likely due to the very low single-dose regimen, this study established the preliminary safety of anti-PD-L1 therapy in non-cancer PWH and provided proof-of-concept that checkpoint blockade could transiently augment HIV-specific immunity in this population. This line of investigation was extended in 2024: in PWH on ART who received low-dose anti-PD-1, a subset of participants showed enhanced HIV-specific immunity and transient viral expression (33). However, significant irAEs were still observed even at low doses, indicating that in tumor-free PWH, the benefit-risk balance of ICI administration requires further exploration.

A substantially more informative study was published in 2025, reporting results from a randomized, double-blind, multicenter, placebo-controlled phase Ib trial of budigalimab, a humanized anti-PD-1 monoclonal antibody, in PWH on suppressive ART without cancer (34). This study went beyond the assessment of virological effects to explore whether PD-1 blockade could confer post-ART-cessation virological benefit. Well-selected participants with favorable immune status and stable virological control were assigned to one of three dose cohorts (2 mg every 4 weeks for two doses, 10 mg every 4 weeks for two doses, or 10 mg every 2 weeks for four doses) along with pooled placebo controls, and underwent a monitored ATI following on-ART dosing. The primary endpoints of safety and pharmacokinetics were met, with acceptable tolerability across all dose levels. The most striking exploratory finding emerged from the highest-dose cohort (10 mg biweekly): six of eleven participants experienced a delay in viral rebound for over six weeks, and two participants maintained virological control throughout the study period, up to 204 and 252 days off ART, respectively. This rate of extended viral control (22.2%) was numerically higher than rates observed in historical cohorts of post-treatment controllers in non-primary infection settings, which typically range from 2.5% to 7.4% (34). None of the participants in the pooled placebo group demonstrated comparable viral control. Nevertheless, given the small sample size, the exploratory nature of the phase Ib endpoints, and the reliance on cross-study historical comparisons, this signal still requires validation in larger confirmatory trials.

However, a subsequent confirmatory trial of budigalimab in PWH (NCT06032546, sponsored by AbbVie) was terminated by the sponsor in October 2025 prior to data publication, underscoring the inherent challenges of advancing early-phase observations into definitive clinical efficacy in non-cancer populations. The budigalimab trial advanced the positioning of ICIs in HIV research from a question of safety to one of potential, albeit limited, post-treatment control. Nevertheless, efficacy clearly varied among individuals, echoing the observations from the CITN-12 multiomic analysis that only those with specific immune characteristics derive meaningful benefit. Although whether functional cure can ultimately be achieved requires more in-depth mechanistic investigations and larger clinical trials, research in non-cancer population has eliminated possible tumor-associated confounding factors, thus providing cleaner clinical settings for the role of ICIs in HIV cure strategies.

### ICIs within the HIV cure scenario

Current HIV cure research largely follows two broad directions. The first targets HIV host cells (CD4+ T cells) or viral integration sites, including CCR5 modification, bone marrow transplantation, and gene editing (e.g., CRISPR/Cas9). The second targets the viral reservoir. Although antiretroviral therapy (ART) can suppress circulating virus, the majority of the virus remains latently silent within host cells, including in tissues such as lymph nodes, and cannot be effectively eliminated owing to the limitations of antiretroviral drugs. Two opposing strategies have been developed to address this reservoir: “shock-and-kill” (reversing latency followed by immune-mediated clearance) and “block-and-lock” (permanently silencing the reservoir to prevent reactivation).

**Figure 3:**
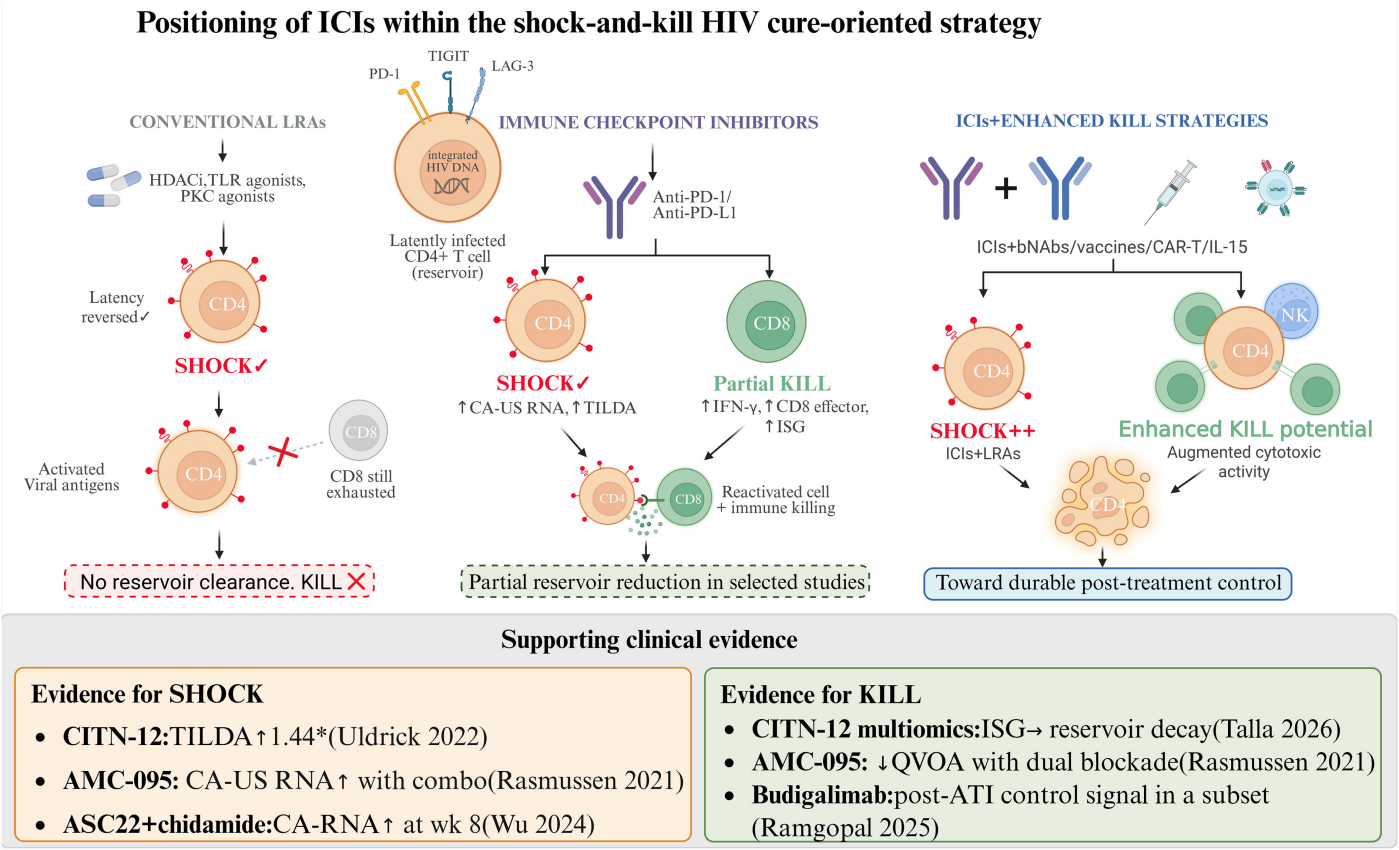
Positioning of immune checkpoint inhibitors within the shock-and-kill HIV cure strategy. The image compares three therapeutic approaches targeting latently infected CD4+ T cells expressing PD-1, TIGIT, and LAG-3. Conventional LRAs such as HDACi, TLR agonists, and PKC agonists can reactivate latent HIV transcription (shock ✓), but do not address T-cell exhaustion; HIV-specific CD8+ T cells remain impaired and cannot clear reactivated cells (kill ✗). ICIs (anti-PD-1/PD-L1 antibodies) induce latency reversal (increased CA-US RNA and TILDA) while reinvigorating exhausted CD8+ T cells (enhanced IFN-γ and effector expansion), producing partial reservoir reduction. Combining ICIs with bNAbs, therapeutic vaccines, IL-15 superagonists, or CAR-T cells is hypothesized to amplify cytotoxic clearance while preserving the latency-reversing effect, representing the most promising path toward reservoir elimination. The bottom panel summarizes supporting clinical evidence from key trials for shock (CITN-12, AMC-095, ASC22+chidamide) and kill effects (CITN-12 multi-omics, AMC-095, budigalimab). bNAbs, broadly neutralizing antibodies; CAR-T, chimeric antigen receptor T cells; CA-US RNA, cell-associated unspliced HIV RNA; CITN-12, Cancer Immunotherapy Trials Network protocol 12; HDACi, histone deacetylase inhibitor; ICIs, immune checkpoint inhibitors; IFN-γ, interferon-gamma; IL-15, interleukin-15; ISG, interferon-stimulated gene; LRAs, latency-reversing agents; PKC, protein kinase C; QVOA, quantitative viral outgrowth assay; TILDA, Tat/Rev-induced limiting dilution assay; TLR, Toll-like receptor; ✓, achieved; ✗, not achieved. Created with BioRender.com.

The shock-and-kill strategy is currently the most extensively studied. Latency-reversing agents (LRAs) can reactivate HIV transcription in latently infected CD4+ T cells, causing these cells to re-express viral antigens and become exposed to the immune system, which then clears the reactivated cells, thereby reducing or potentially eradicating the reservoir. In an ideal scenario, ICIs would play a unique role, as they theoretically possess the capacity to fulfil both components of this strategy simultaneously: activating PD-1-expressing reservoir cells to reverse latency (“shock”) while reinvigorating exhausted HIV-specific CD8+ T cells to enhance immune clearance (“kill”). **Figure 3** illustrates the conceptual positioning of ICIs within the shock-and-kill paradigm, comparing them with conventional latency-reversing agents and rational combination strategies.

In practice, however, this strategy still faces major challenges, and the difficulty lies not in the “shock” but in the “kill.” The readouts used to assess shock (latency reversal) include CA-US RNA, TILDA, and the RNA : DNA ratio, whereas those used to assess the kill include total HIV DNA, integrated HIV DNA, QVOA, and IPDA — measures that reflect whether infected cells are genuinely reduced. Multiple studies indicate that IPDA offers the best accuracy and most faithfully reflects true changes in reservoir size. Although IPDA retains some error owing to methodological limitations and the high mutation rate of HIV, it remains the current benchmark for quantifying intact and defective proviral DNA. Importantly, an increase in shock markers does not equate to a reduction in the reservoir.

Among the existing evidence for ICIs, most studies indicate that the shock is readily achieved: TILDA increased 1.44-fold in the CITN-12 trial (Uldrick et al.; P = 0.008); CA-HIV RNA increased in the case series by Peng et al. (P = 0.049); CA-HIV RNA rose at week 8 with ASC22 plus chidamide; CA-US RNA increased in a subset of participants in the study by Lau et al.; and CA-US RNA also increased in the combination arm of AMC-095 (Rasmussen et al.). These data confirm that latency reversal can be achieved with ICIs. Yet among these numerous studies, genuine reservoir clearance was observed in very few instances, and almost all are limited by small sample sizes and considerable heterogeneity.

This asymmetry — robust latency reversal but scarce reservoir reduction — represents the central gap in the field. The root cause is that activation does indeed occur, but the reactivated cells are not eliminated. Chronic HIV infection drives T cells into a state of exhaustion. Although PD-1 blockade can transiently reinvigorate exhausted T cells, it cannot reprogram them into durable memory T cells (TMEM). Reinvigorated exhausted T cells only transiently reacquire some functional features of effector T cells (TEFF), while their underlying epigenetic fate remains unchanged; under persistent antigenic stimulation, this transient effect again progresses toward exhaustion. The distinct and stable epigenetic landscape of exhausted T cells fundamentally explains this “effective but not durable” phenomenon. This is precisely why many clinical trials have failed to achieve lasting immune responses. Consequently, achieving durable effects in the future will require combination strategies, such as reducing persistent antigenic stimulation, providing vaccine or cytokine support, modulating cell differentiation, and combining these with reservoir-clearance approaches, to make durable immunity and ultimately an HIV cure achievable.

### ICIs as a component of combination cure strategy

The cellular foundation for this dual role was established by Fromentin et al. (19), who demonstrated that CD4+ T cells expressing PD-1, TIGIT, and LAG-3, particularly those co-expressing all three markers, are major hosts of persistent HIV and the inducible reservoir during ART. Triple-positive CD4+ T cells were enriched 8.2-fold for integrated HIV DNA compared with bulk CD4+ T cells, and cells expressing at least one of these markers accounted for a median of 76% of the inducible reservoir. This finding provided the core rationale for subsequent investigations of ICIs as reservoir-targeting agents. Combined with the clinical demonstration by Uldrick et al. (22) that anti-PD-1 can induce latency reversal *in vivo*, and the preclinical observation by Pereira Ribeiro et al. (25) that single-pathway blockade with anti-IL-10 alone was insufficient for stable viral control and that effective outcomes likely depend on multi-pathway immune coordination, investigators proceeded to explore combination regimens incorporating ICIs.

A phase II single-arm study evaluated the combination of ASC22, an anti-PD-L1 antibody, with chidamide, a subtype-selective HDACi, in ART-suppressed PWH (24). The design rationale was that chidamide would serve as a potent LRA, while ASC22 would enhance HIV-specific immunity to clear the reactivated cells. Among the 15 participants who completed the study, CA-HIV-RNA levels did show a significant increase from baseline at week 8 which returned to baseline following discontinuation, confirming the latency-reversing effect of the combination. However, total HIV DNA was only transiently elevated at week 4 (P = 0.014), integrated HIV DNA did not significantly differ from baseline at any time point, and HIV-specific CD8+ T cell function was not meaningfully enhanced (24). The combination failed to provide evidence for effective reservoir clearance, indicating that the “kill” component requires further optimization.

Beyond the combination of ICIs with HDACi, several other combination strategies are under active investigation or theoretical consideration. The preclinical success of dual IL-10/PD-1 blockade (25) has generated interest in clinical translation of multi-pathway immunomodulation. With respect to combining ICIs with therapeutic vaccination, preliminary human evidence is now available. Marin et al. found that in PWH who received early ART and a therapeutic vaccine, PD-1 blockade significantly enhanced vaccine-induced HIV-specific CD8+ T-cell function, whereas TIM-3 blockade had no apparent effect (35). This suggests that PD-1 pathway blockade may serve as an effective adjuvant for therapeutic vaccines, enhancing the vaccine response. Furthermore, ICIs combined with broadly neutralizing antibodies (bNAbs), bispecific or trispecific antibodies, therapeutic vaccines, IL-15 or IL-15 superagonists, and CAR-T or TCR-T cell therapies represent theoretically attractive approaches, where ICIs could first relieve immune exhaustion followed by various “kill” strategies that enhance cytotoxic elimination of reservoir cells. However, clinical data for such combinations remain unavailable, and their efficacy and safety await validation in well-designed trials (21, 36–38).

### Changes in the proviral landscape following ICIs

Recent studies indicate that the effects of ICIs on the HIV reservoir may extend beyond quantitative modulation of reservoir size to encompass detectable changes in the qualitative composition of proviral sequences. As noted above, single-genome sequencing analysis of the individual with persistent low-level viremia in the CITN-12 study demonstrated that this viremia did not originate from abnormal expansion of a single infectious clone but rather reflected broad induction of expression from the reservoir following latency reversal (22).

Guiraud and colleagues (39) further advanced this line of investigation through matched integration site and proviral sequencing analysis in three PWH who received ICIs for cancer within the ANRS CO-24 OncoVIHAC cohort (two treated with anti-PD-1/PD-L1 and one with combined anti-PD-1/CTLA-4). Although total CA HIV DNA did not change significantly, and the genomic and epigenetic features of integration sites were not appreciably affected, ICIs were associated with non-random changes in proviral sequence composition: all treatment regimens were linked to a reduced proportion of proviruses containing *pol* frameshifts, while patients receiving anti-PD-1 or combined anti-PD-1/CTLA-4 exhibited an increased proportion of proviral sequences harboring *gag* frameshifts and premature stop codons, consistent with a *gag*-driven immune clearance mechanism. Additionally, the patient receiving combination therapy lost an HIV clone integrated in the *NIN* oncogene that had represented 17% of all pretreatment sequences, and a modest reduction in reservoir diversity was also observed (39).

These findings carry important theoretical implications. Even when total reservoir size does not change significantly by quantitative measures, selective alterations observed at the sequence level indicate that immune-mediated clearance is operative. The impact of ICIs on the reservoir may follow a progressive course: initial activation of the inducible reservoir and release of virus, followed by gradual remodeling of proviral sequence composition and clonal structure as immune effector functions are enhanced. This suggests that the effects of ICIs on the HIV reservoir are not limited to the conventional shock-and-kill framework, but also involve deeper molecular and genetic changes that may inform future therapeutic strategies.

### Safety profiles

The safety of ICIs in people with HIV (PWH) has long been a matter of close concern, and it was precisely this concern that initially led to the exclusion of this population from cancer immunotherapy trials (3). However, since 2015, accumulating clinical evidence - from early case reports to prospective clinical trials - has progressively confirmed the safety of these agents in PWH (4–10). In the early stages, Davar et al., Heppt et al., and Bertin et al. reported that ICIs were well tolerated in PWH with advanced cancers, with no opportunistic infections or serious immune-related adverse events observed during follow-up (4–6). These studies collectively demonstrated that ICIs are not contraindicated in this population, laying the foundation for larger-scale clinical evaluation.

With regard to immune-related adverse events (irAEs), multiple systematic reviews have shown that the incidence of grade 3 or higher irAEs in PWH is approximately 8-10%, comparable to HIV-seronegative populations, with a similar spectrum of cutaneous, gastrointestinal, hepatic, and endocrine toxicities (8, 9). However, the safety assessment of ICIs in PWH should not focus solely on the incidence of irAEs; attention must also be paid to the secondary risks arising from their management. ICIs are inherently immunostimulatory, yet the standard management of moderate-to-severe irAEs relies on glucocorticoids, and refractory cases may require immunosuppressive agents such as TNF-α antagonists. These immunosuppressive interventions may paradoxically increase the risk of opportunistic infections, including reactivation of latent tuberculosis, Pneumocystis pneumonia (PCP), invasive aspergillosis, cytomegalovirus (CMV) disease, and herpesvirus reactivation (12, 40, 41). Pharmacovigilance data indicate that most ICI-associated infections occur within the first few months after treatment initiation, with significantly elevated reporting rates for PCP and bacterial pneumonia; the risk is further increased when combined immunosuppressive therapy is used (41). Therefore, in PWH with incomplete immune reconstitution, the risk of infection from excessive immunosuppression must be carefully weighed when managing irAEs. Notably, a fatal case of HHV-8-associated polyclonal B-cell lymphoproliferative disorder was reported in a patient with Kaposi sarcoma enrolled in the CITN-12 study, indicating that severe and unpredictable complications may still arise in the setting of HIV-related immune dysregulation (7).

IRIS-like events have been exceedingly rare in the context of ICI use, likely because enrolled participants had well-suppressed viremia. The predominant factor in IRIS prevention is immune recovery established during early ART, rather than the immunological effects of ICIs themselves (42). Across the vast majority of reported studies, ICI administration did not compromise HIV virological suppression; plasma HIV RNA and CD4+ T-cell counts remained stable, and no cases of virological failure attributable to ICI therapy were observed (7, 8, 22, 32–34). Furthermore, PD-1, PD-L1, and CTLA-4 antibodies, as large-molecule proteins cleared through proteolytic catabolism, are not expected to have clinically significant pharmacokinetic interactions with antiretroviral agents. However, pharmacodynamic interactions at the immunological level have not been systematically evaluated and warrant further investigation (7, 8).

## Emerging targets beyond PD-1 and CTLA-4

While PD-1 and CTLA-4 have dominated the landscape of checkpoint blockade research in both oncology and HIV, accumulating evidence implicates additional immune checkpoint receptors in HIV persistence, raising the possibility that targeting these molecules may provide complementary or synergistic benefits. Joller et al. (14) systematically reviewed the differential functions of LAG-3, TIM-3, and TIGIT, the next generation of immune checkpoint receptors currently being harnessed in clinical oncology, and demonstrated that these molecules serve distinct rather than redundant roles across different cell types, activation stages, and tissue environments: LAG-3 primarily suppresses early immune responses, helping maintain a low-reactivity state; TIGIT operates through regulatory T cell (Treg), regulatory B cell (Breg), and dendritic cell networks to reinforce an immunosuppressive environment; and TIM-3 preferentially regulates terminally exhausted T cells and innate immune cell function. These distinct suppressive programs collectively contribute to the immune dysfunction observed in chronic HIV infection.

TIGIT. TIGIT is co-expressed with PD-1 on CD4+ T cells harboring latent HIV, and this co-expression is maintained during suppressive ART. As previously discussed, Fromentin et al. (19) demonstrated that CD4+ T cells co-expressing PD-1, TIGIT, and LAG-3 were enriched 8.2-fold for integrated HIV DNA compared with bulk CD4+ T cells, and that cells expressing at least one of these markers accounted for a median of 76% of the inducible reservoir. In the oncology field, several anti-TIGIT antibodies (e.g., tiragolumab, vibostolimab) are in advanced clinical trials, although none have been evaluated in the HIV setting to date.

LAG-3. LAG-3 expression is upregulated on T cells during HIV infection and cooperates with PD-1 in marking reservoir cells. Importantly, pre-ART expression of PD-1, LAG-3, and TIM-3 on CD4+ and CD8+ T cells has been identified as a predictor of time to viral rebound following treatment interruption (12). The anti-LAG-3 antibody relatlimab has been approved in combination with nivolumab for unresectable or metastatic melanoma, establishing clinical precedent for dual checkpoint blockade involving this target.

TIM-3. In progressive HIV infection, TIM-3 expression is upregulated on HIV-specific CD8+ T cells. TIM-3-positive T cells exhibit impaired cytokine production and proliferative capacity, and blockade of the TIM-3 signaling pathway has been shown to restore proliferation and enhance cytokine production in HIV-specific T cells *ex vivo* (43). Multiple anti-TIM-3 antibodies are currently in phase I/II clinical trials in oncology.

The stage-specific expression patterns of these next-generation checkpoints carry important implications for HIV cure strategies: targeting different checkpoints may engage different subsets of the reservoir and different populations of immune effector cells. Since TIGIT, LAG-3, and TIM-3 operate through distinct signaling mechanisms and regulate different aspects of immune function, they may offer more favorable safety profiles than PD-1 or CTLA-4 blockade when used alone, while potentially achieving synergistic antiviral effects in rational combinations. However, multi-target checkpoint blockade may also increase the risk of severe irAEs due to excessive immune activation, necessitating careful evaluation of safety in well-designed clinical trials. Clinical data on these novel targets in the HIV setting remain absent, representing a gap of considerable research interest.

## Future directions and challenges

Current evidence has demonstrated that ICIs can serve as promising tools in addressing HIV persistence; however, numerous challenges remain before they can be translated into practical cure strategies. The most formidable of these is the “kill” component of shock-and-kill - how to achieve genuine clearance of the reactivated reservoir and thereby realize an HIV cure. Previous studies have shown that single-agent ICIs are unlikely to accomplish this, primarily because the epigenetic fate of exhausted T cells is difficult to reverse: even after transient reinvigoration, these cells cannot be reprogrammed into durable memory T cells and therefore fail to exert sustained clearance (44, 45). As a result, the “kill” remains limited. How to deliver ICIs more safely, how to identify responders through biomarker screening, and how to enhance clearance through combination strategies are therefore the foremost priorities in current ICI research. All of these efforts fundamentally revolve around enhancing and sustaining the “kill,” and among them, combination strategies represent the key to overcoming the current bottleneck.

Safer dosing regimens for non-cancer populations. ICIs are generally well tolerated in cancer patients, but their safety in otherwise healthy PWH on effective ART remains uncertain. The budigalimab trial demonstrated that doses far below those used in oncology were well tolerated in tumor-free PWH. However, the 2 mg dose did not sustain PD-1 receptor saturation, and the antiviral signal was confined to the highest-exposure regimen, indicating a lower bound on the exposure required for activity (34). Future studies should therefore explore optimal ICI dosing in PWH; this could minimize immune-related toxicity while preserving the shock-and-kill effect. In parallel, additional types of checkpoint inhibitors should be developed, including next-generation PD-1 inhibitors, novel inhibitors targeting other exhaustion-associated molecules, bispecific antibodies, and antibodies engineered to reduce Fc effector function, thereby broadening the therapeutic window for use in the HIV population and enabling larger clinical trials.

Biomarker-guided identification of responders. Across previous studies, there has been considerable heterogeneity, and the populations sensitive to ICIs have varied; only a subset of individuals demonstrated meaningful virological and immunological responses. To address this, the CITN-12 cohort conducted a multi-omics analysis to identify responder characteristics, identifying sustained ISG activation, induction of antiviral restriction factors, and early effector CD8+ T-cell expansion as correlates of reservoir decay (29). Other studies have also shown that the CD4+/CD8+ is a potential predictor of latency reversal (22). These markers hold promise for the prospective screening of responders. However, such studies remain scarce, and future efforts should focus on developing and validating biomarker panels to enable precision therapy, prospective identification of individuals most likely to benefit from ICIs, and a reduction in unnecessary drug exposure among non-responders.

Optimization of combination strategies. Given the limitations of ICIs in the “kill” component, combination strategies can compensate for this shortcoming. Current regimens under investigation include ICIs combined with broadly neutralizing antibodies (bNAbs), ICIs combined with therapeutic vaccines, and ICIs combined with next-generation LRAs. The preclinical success of IL-10/PD-1 dual blockade, clinical evidence for enhanced efficacy with PD-1/CTLA-4 combination therapy, and the demonstration that PD-1 blockade can enhance vaccine-induced CD8+ responses all provide preliminary evidence that multi-pathway combinations are more effective than monotherapy (23, 25, 35). Accordingly, “ICIs plus therapeutic vaccines” and “multi-pathway immune modulation” may represent the most promising directions for breakthroughs in the coming years. Regarding the optimal sequence of these interventions, no definitive guidance is currently available; however, one reasonable hypothesis is that ICIs should be administered before ATI to activate latent virus and prime the immune system, followed by more effective “kill” strategies to control rebound viremia. This sequencing question warrants systematic investigation. Addressing these issues will also require standardized reservoir quantification methods such as the intact proviral DNA assay (IPDA) (46–48), as well as careful ethical consideration of ATI-based endpoints. The latest ATI consensus guidelines emphasize that, while maintaining a safety floor, greater flexibility in ATI design should be adopted to accommodate different scientific questions, geographic settings, and study populations. Compared with earlier guidelines, this signals that future trial designs may become more flexible, and that trials evaluating combination strategies will become increasingly common (49, 50).

Bridging the gap to functional cure. The most fundamental challenge at present is that ICIs can perturb the reservoir but achieving functional cure - sustained virological control in the absence of ART - remains a distant goal. Even in the budigalimab trial, where 2 of 11 high-dose recipients maintained viral suppression for over 200 days, the majority of participants experienced viral rebound (34). How to achieve deeper and more durable reservoir reduction through more intensive or more precisely targeted immunotherapies is therefore the central question. At this stage, achieving this goal through ICIs alone appears unlikely; this is not only because of T-cell exhaustion but may also be constrained by intrinsic biological barriers, including anatomical sanctuary sites, proviral integration into transcriptionally silent genomic regions, and clonal expansion of infected cells (21, 39). ICIs should instead be combined with other therapies to enhance the “kill”; only then may a functional cure become achievable.

## Conclusions

ICIs have recently emerged as a promising class of immunotherapeutic agents with the potential to address both the inflammatory dysregulation and viral persistence in chronic HIV infection. Preclinical evidence from NHP models, clinical observations in PWH with cancer, and early-phase trials in non-cancer populations collectively demonstrate that ICIs, particularly those targeting the PD-1/PD-L1 axis, can reverse HIV latency *in vivo*, enhance HIV-specific immune responses, and in some cases contribute to post-treatment virological control. The dual capacity of ICIs to serve as both latency-reversing agents and immune enhancers has positioned them uniquely within the HIV cure paradigm. Combination approaches, e.g. coupled with anti-CTLA-4, or HDACi, or agents targeting other pathways, appear to offer superior efficacy over single-agent regimens (22–25, 34, 39). Nevertheless, current evidence remains limited by small sample sizes and substantial heterogeneity across studies, and the mechanistic investigation remains quite preliminary. Critical priorities for the field include the systematic evaluation of rationally designed combination strategies, development of safer dosing regimens, construction of biomarker panels to predict treatment response, standardization of reservoir measurement assays, and conduct of adequately powered randomized controlled trials. With continued investment in translational research and well-designed clinical studies, ICIs hold genuine potential to contribute to the ultimate goal of a functional cure for HIV infection.
